# The Oleates

**Published:** 1885-07

**Authors:** 


THE OLEATES: AN INVESTIGATION INTO THEIR NATURE AND ACTION. *By* JOHN V. SHOEMAKER, A. M., M. D., *Lecturer on Dermatology at the Jefferson Medical College, etc., Philadelphia.* F. A. DAVIS, ATT'Y. *1885.*

The author of this little book has devoted so much time to a study of the therapeutical effects of the several oleates that he may be said to have succeeded in associating his name with the subject. There can be little question but that several of the oleates, more particularly those of zinc and copper, should enjoy a position in the therapeutics of external treatment second only to that in which the oleates of lead and mercury were placed long before the number of combinations of this sort had reached the present figure. Certainly Attfield and Marshall could not have suspected, when they first wrote in connection with the subject, the extent to which the usefulness of the two oleates named would be eventually acknowledged.

Dr. Shoemaker's little book will prove useful to those who are interested in this subject, for they will find the advertisement of a well-known drug house printed after the conclusion of the pages written by the author, in which oleates manufactured and sold by the former are duly enumerated.

The general style of the author may be illustrated by the following extracts:

"Although this had been disputed by some, who, however, constitute no medical authority, having a commercial interest only in the sale of their goods, and for pharmaceutical reasons lay more stress on their handsome appearance than on their utility, I think this matter should rest with the physician to decide rather than to the biased vendor." P. 61. (*Sic.*)

Again: "The communications that have appeared from the latter class speak of their action disparagingly, and their effects as problematical, but are devoid of research, which appears when they speak of quinine oleate being limited to inunctions for its systemic impression, showing an absence of practical experience or proper deductions from their results." P. 110.

